# Using saturation rational function models to calculate yield adjustment factors across varied milking frequencies

**DOI:** 10.3168/jdsc.2024-0720

**Published:** 2025-08-20

**Authors:** Xiao-Lin Wu, John Cole, Asha M. Miles, Paul M. VanRaden

**Affiliations:** 1 Council on Dairy Cattle Breeding, Bowie, MD 20716; 2 Department of Animal and Dairy Sciences, University of Wisconsin–Madison, Madison, WI 53706; 3 Department of Animal Sciences, Donald Henry Barron Reproductive and Perinatal Biology Research Program, and the Genetics Institute, University of Florida, Gainesville, FL 32608; 4 Department of Animal Science, North Carolina State University, Raleigh, NC 27607; 5 USDA Animal Genomics and Improvement Laboratory, Beltsville, MD 20705

## Abstract

Milking frequency significantly affects milk yield in dairy cows, with higher frequency generally leading to greater lactation yields. The increase follows a nonlinear pattern, showing diminishing returns and eventually reaching saturation as milking frequency rises. This study introduces a polynomial rational function model to derive yield adjustment factors across different milking frequencies. Formulated as a ratio of 2 polynomials, this model has 3 parameters to capture the initial increase in yield and the saturation rate, offering enhanced flexibility across various milking frequencies. We compared its performance to a recently proposed exponential rational function model. Both models demonstrated a good fit to varying milking frequency data up to 10× and satisfactorily predicted yield adjustment factors for milking frequencies where data were absent. The polynomial rational function model exhibited a higher accuracy (root mean square error [RMSE] = 0.004; R^2^ = 0.999), achieving greater accuracy across a broader range of varied milking frequencies, compared with the exponential rational function model (RMSE = 0.011; R^2^ = 0.994). Nevertheless, the latter model proved more robust to limited data coverage of milking frequencies. This study also evaluated the strategy of leveraging 2× milking data to derive yield adjustment factors across different frequencies. However, caution is advised when extrapolating far beyond the data-supported frequency range.

## Highlights

• The single-parameter exponential RF model shows greater robustness with limited data.
• The 3-parameter polynomial RF model offers enhanced model flexibility.
• Both models provide good model fitting and accurate predictions with data support.
• Deriving adjustment factors by leveraging 2× milking records is evaluated.

## Graphical Abstract

**Figure:**
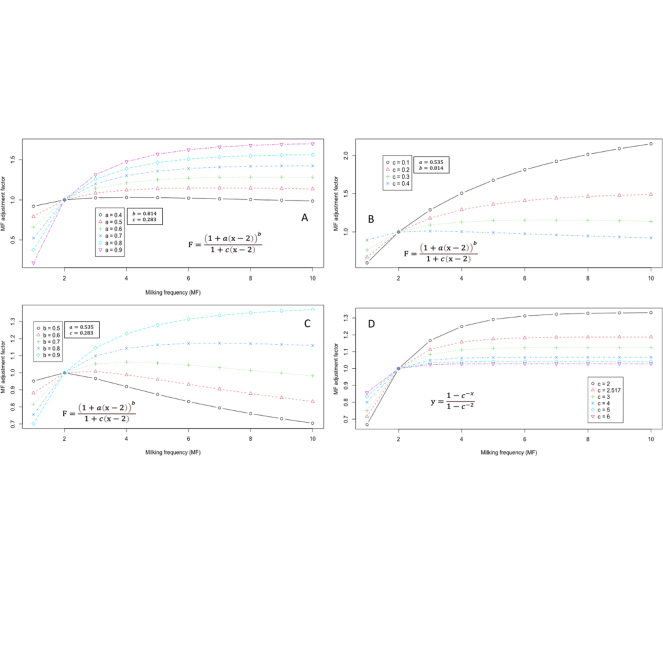
**Summary:** Milking frequency significantly affects milk yield in dairy cows, with higher frequency generally resulting in increased lactation milk yields. The increase follows a nonlinear pattern, with diminishing returns as milking frequency rises, and eventually reaches a saturation point at higher milking frequencies. This study compared 2 saturation rational functions (RF) to derive yield-adjustment factors across different milking frequencies.

Milking frequency significantly influences a cow's milk production (Hale et al., 2003). Biologically, milk production occurs more rapidly when the mammary gland is less full and slows as it fills. Hence, more frequent milking leads to regular udder emptying, which signals the body to increase milk synthesis through a supply-and-demand mechanism. As a result, cows milked 3 or more times daily typically produce more milk than those milked twice daily. However, because these differences in yield arise from environmental rather than genetic factors, adjustments are applied in genetic evaluations to ensure fair comparisons among different milking frequencies.

In the United States, for instance, the genetic merit of animals has been assessed using mature-equivalent, twice-daily milking, 305-d lactation yields (**305-ME**) based on a multiple-trait animal model (Wiggans et al., 1988; Wiggans and VanRaden, 1989; VanRaden et al., 1995). Effective June 12, 2024, the standardized yield records are now adjusted to 36 mo, referred to as the 305-AA yield, instead of 305-ME (https://uscdcb.com/august-2024-evaluations-whats-new/). The new adjustments accounted for the differences due to age, parity, and seasons. Still, the requirement for standardizing lactations to a 2× milking frequency basis remains unchanged.

Lactation yields from cows milked *k* times per day (*k*×) are adjusted to a 2× basis by the following formula:

[1] $$y_{2\times }=\frac{y_{k\times }}{1+RI\times \left(\frac{DIM_{k\times }}{DIM}\right)},$$

where *y*_2×_ is the 2× equivalent lactation yield, *y*_k×_ is the yield from the *k*× milkings daily, RI is the relative increase in milk production, DIM*_k_*_×_ is the number of days with *k*× milkings, and DIM is the total days in milk. If the same *k*× milkings were conducted for the entire lactation period, the above formula simplifies to

[2] $$y_{2\times }=\frac{y_{k\times }}{1+RI}.$$

This traditional approach derives a relative increase (1 + **RI**) factor based on real-world data, thus making it grounded in observed production patterns. For instance, the estimated RI for 3× milkings relative to 2× milkings ranged from 1.49 (for cows calving at 18 mo) to 1.04 (for cows calving at 48 mo) and from 1.20 (for 2-yr-old cows) to 1.15 (for 4-yr-old cows; Norman et al., 1974). However, milking data for frequencies greater than 3× are often not available. Statistically, these discrete RI values represent degenerated distributions, which do not automatically project to higher milking frequencies.

Recently, VanRaden et al. (2023) proposed a saturation rational function as a ratio of 2 exponential components, referred to as saturation exponential rational function (**ERF**):

[3] $$F=\frac{1-c^{-x}}{1-c^{-2}}.$$

The numerator in Equation [3] represents the difference between 1 and an exponential decay term, *c*^−^*^x^*, where *c* > 1 is a positive constant and *x* denotes milking frequency. The denominator, 1 − *c*^−2^, is a scaling constant for a given *c*-value, where 2 represents 2× milking, thus adjusting the function to normalize the behavior of the numerator relative to 2× milking as the baseline. For *c* > 1, the function is monotonic, where *c*^−^*^x^* decreases exponentially as milking frequency (*x*) rises. The milk yield adjustment factor *F* corresponds to 1 + RI in Equation [2]. The value of *F* is less than 1 if *x* < 2 and exceeds 1 for *x* > 2. As milking frequency increases, *c*^−^*^x^* approaches 0 asymptotically, causing *F* to approach $\frac{1}{1-c^{-2}}$ (see graphical abstract for *c* = 2; panel D). This 1-parameter model is easy to compute but lacks the flexibility to capture complex growth or decay patterns.

This paper introduces a saturation polynomial rational function (**PRF**) model for deriving milk yield adjustment factors due to varied milking frequencies. The PRF model also assumes that increased milking frequency stimulates sustained milk production, leading to higher yields. The increase in yield with each additional milking is nonlinear, showing diminishing returns as frequency rises. However, unlike ERF, this new model allows a decline in the rate of increase after the saturation point is reached, likely reflecting physiological limitations and adaptations in the cow. In practice, several factors may contribute to the reduced rate of milk production after saturation is reached. For example, the mammary tissue can experience fatigue from higher milking frequencies over time, diminishing its capacity to sustain peak production, possibly due to cellular stress or age-related changes in the udder. Also, hormonal signals that regulate milk synthesis and secretion may decrease after saturation, leading to a slowdown in milk production. Possibly, the physical capacity of the udder is limited, and there may be a biological ceiling on milk production, regardless of how frequently the cow is milked.

A reasonable starting point is a modified polynomial model where the increase in yield with additional milking frequency is proportional to the baseline milk yield but at a diminishing rate:

[4] $$y_{t}=y_{2\times }{\left(1+a\left(\frac{24}{t}-2\right)\right)}^{b},$$

where *y_t_* is the daily milk yield at a milking interval of *t* in hours, *y*_2×_ is the twice-daily milk yield, *a* represents the rate of change in milk yield as the milking interval is shortened or lengthened compared to twice-daily milking, and *b* is the diminishing-return parameter (typically 0 < *b* < 1, indicating that the increase diminishes as the milking frequency rises).

Mathematically, Equation [4] assumes that the milk yield keeps increasing indefinitely as milking frequency increases. In reality, milk yield tends to plateau (saturate) or even decrease as the milking frequency increases. To account for this feature, we divided the right-hand side of Equation [4] by a term equaling $1+c\left(\frac{24}{t}-2\right):$

[5] $$y_{t}=y_{2\times }\times \frac{{\left(1+a\left(\frac{24}{t}-2\right)\right)}^{b}}{1+c\left(\frac{24}{t}-2\right)}.$$

The denominator introduces saturation, preventing indefinite growth by incorporating a parameter, *c*, that controls the rate at which the yield plateaus or decreases as milking frequency increases. This function can be viewed as extending Equation [2] by replacing 1 + RI with

$$\frac{{\left(1+a\left(\frac{24}{t}-2\right)\right)}^{b}}{1+c\left(\frac{24}{t}-2\right)}.$$

Expressing Equation [5] as a milk yield adjustment factor for varied milking frequencies relative to twice-daily milking gives

[6] $$F=\frac{y_{t}}{y_{2\times }}=\frac{{\left(1+a\left(x-2\right)\right)}^{b}}{1+c\left(x-2\right)},$$

where $x=\frac{24}{t}.$ This new model has 3 parameters, offering flexibility to capture different growth patterns as milking frequency increases. As illustrated in the graphical abstract (panels A, B, and C), adjusting the values for *a*, *b*, and *c* allows the model to adapt to various scenarios, such as rapid initial growth with early saturation or slower growth with delayed saturation.

To illustrate the effectiveness of these two rational functions, we first fitted them to data following VanRaden et al. (2023). In New Zealand, an estimated yield of 75% was reported for 1× milkings relative to 2× milkings (Murphy et al., 2023; VanRaden et al., 2023), while the US standard for 2× milkings has consistently been set at 1.0. Furthermore, official data from Iowa State University (Ames, IA) suggested an average adjustment factor of 1.11 for 3× milkings. For 4× or more frequent milkings, the actual field data are unavailable. Instead, we used the predicted adjustment factor values by VanRaden et al. (2023) as proxy “actual” data for milking frequencies ranging from 4× to 10×. The purpose was to demonstrate the method, not to obtain the actual predictions of adjustment factors. The models were fitted under two scenarios (Table 1). In the first scenario (**S1**), we assumed having observed the relative increment factor values in milk yields up to 10× daily milkings and then conducted an in-sample validation. In the second scenario (**S2**), we assumed known relative increment factor values up to 5× daily and predicted adjustment factors for unknown high-frequency (6×–10×) milkings.

**Table 1:** Estimated model parameters and milking yield adjustment factors for varied milking frequencies relative to twice-daily milking based on 2 saturation rational function models^1^

Model	Data	ERF	PRF	Data	ERF	PRF
Parameter						
*a*			0.54 (0.01)			0.56 (0.32)
*b*			0.81 (0.03)			0.56 (1.20)
*c*		2.52 (0.03)	0.28 (0.02)		2.57 (0.08)	0.16 (0.48)
Milking frequency						
1	0.75	0.72	0.75	0.75	0.72	0.75
2	1.00	1.00	1.00	1.00	1.00	1.00
3	1.11	1.11	1.10	1.11	1.11	1.10
4	1.16	1.16	1.15	1.16	1.16	1.15
5	1.18	1.18	1.19	1.18	1.18	1.18
6	1.19	1.18	1.19		1.18	1.19
7	1.19	1.19	1.19		1.19	1.19
8	1.19	1.19	1.19		1.19	1.19
9	1.19	1.19	1.19		1.19	1.19
10	1.19	1.19	1.19		1.19	1.19

1 ERF = exponential rational function model proposed by VanRaden et al. (2023); PRF = polynomial rational function proposed in the present study. Data were taken from VanRaden et al. (2023). Data and results are presented as rounded to the second decimal. Please refer to model (3) or (5) for the interpretation of parameters *a*, *b*, and *c*.

The estimated model parameters and predicted adjustment factors for varying milkings using the two models are shown in Table 1. For the ERF model, the estimated *c*-values were ∼2.52 and 2.57, respectively, across both scenarios. The ERF, being a single-parameter model, is more sensitive to the initial growth phase, where changes are rapid, than to the later phase after saturation. Therefore, missing data beyond 5× milkings did not have a significant effect on the PRF model. For the PRF model, the estimated *a*-values were relatively consistent between both scenarios, ranging from 0.54 to 0.56. This parameter controls the initial growth rate, with a larger *a*-value corresponding to faster growth at lower milking frequencies. However, the estimated *b* and *c*-values showed significant differences. The *b*-parameter affects the curvature of growth; a larger *b*-value results in a steeper initial increase followed by a more abrupt deceleration. The *c*-parameter influences the saturation point, with a larger *c*-value leading to earlier saturation. In this analysis, milking data were available for up to 5× milkings in S1 and S2, providing sufficient information for the initial growth estimation in both scenarios. Consequently, fitting the PRF model to the data, resulted in similar *a*-estimates regardless of the data availability beyond 5× milkings. However, the growth curvature and saturation are shaped more crucially at higher milking frequencies, which lacked actual data support under S2. The estimates of the *b*- and *c*-parameters with PRF varied substantially between S1 and S2.

Despite these differences, both models performed well in fitting the data known for up to 10× milkings, and they accurately predicted the adjustment factors where the data were unavailable. The PRF model had a lower root mean square error (**RMSE**; RMSE = 0.004) and a higher R^2^ (0.999) compared to the ERF model (RMSE = 0.011; R^2^ = 0.994). Mathematically, an ERF is monotonical, whereas a PRF model can capture more varied patterns of milk yield changes after saturation.

In practice, collecting and comparing complete lactation yields across various milking frequencies poses significant practical challenges due to the time and expense involved, particularly when involving high milking frequencies (e.g., between 4× and 10×). However, 2× and 3× milking data are more commonly accessible. A practical question arises: Can they be effectively used to derive adjustment factors for different milking frequencies? We addressed this question using a dataset of 15,888 Holstein milking records (7,544 a.m. and 7,544 p.m.) collected from 3,717 animals, randomly sampled from 23 herds across 11 US states between 2006 and 2009 (Wu et al., 2023). The records covered the first 3 lactations (39.8% first, 59.4% second, and 0.8% third lactation) and represented 4 of the 5 US geographical climate regions (https://codes.iccsafe.org/content/IECC2021P1/chapter-3-ce-general-requirements).

Data cleaning excluded outliers and retained morning and evening milking records for milking intervals of approximately between 6 and 18 h. To derive lactation yield adjustment factors across varied milking frequencies, daily milk yields were re-estimated hypothetically. For example, if the milking interval for a cow was 12 or 8 h, the daily yield was calculated as 2 or 3 times its partial (a.m. or p.m.) yield, respectively. For cows with different milking intervals, such as *t* = 10 h, the milking frequency was $\frac{24}{t}=2.4,$ and a corresponding daily yield was assigned as 2.4 times the partial yield. Figure 1 shows the distribution of recalculated daily yields by 30-min milking interval classes, where hypothetical milking frequencies ranged approximately between 1.3× and 3.8×. Quadratic smoothing of the data showed a gradual increase in average daily yield from 1.3× to 3.1× milking, followed by a decrease beyond that point. The drop at around 3.1× milking could result from a limited number of milking records rather than being intrinsic. Previous studies, including that of VanRaden et al. (2023), suggested that the change in yield tended to stabilize for milking frequencies of 4× or more frequent milkings, albeit at a minimum increase compared to 2× milkings. Additionally, noticeable data variations were observed for <2× milkings. The underlying reasons remain unknown.

**Figure 1:**
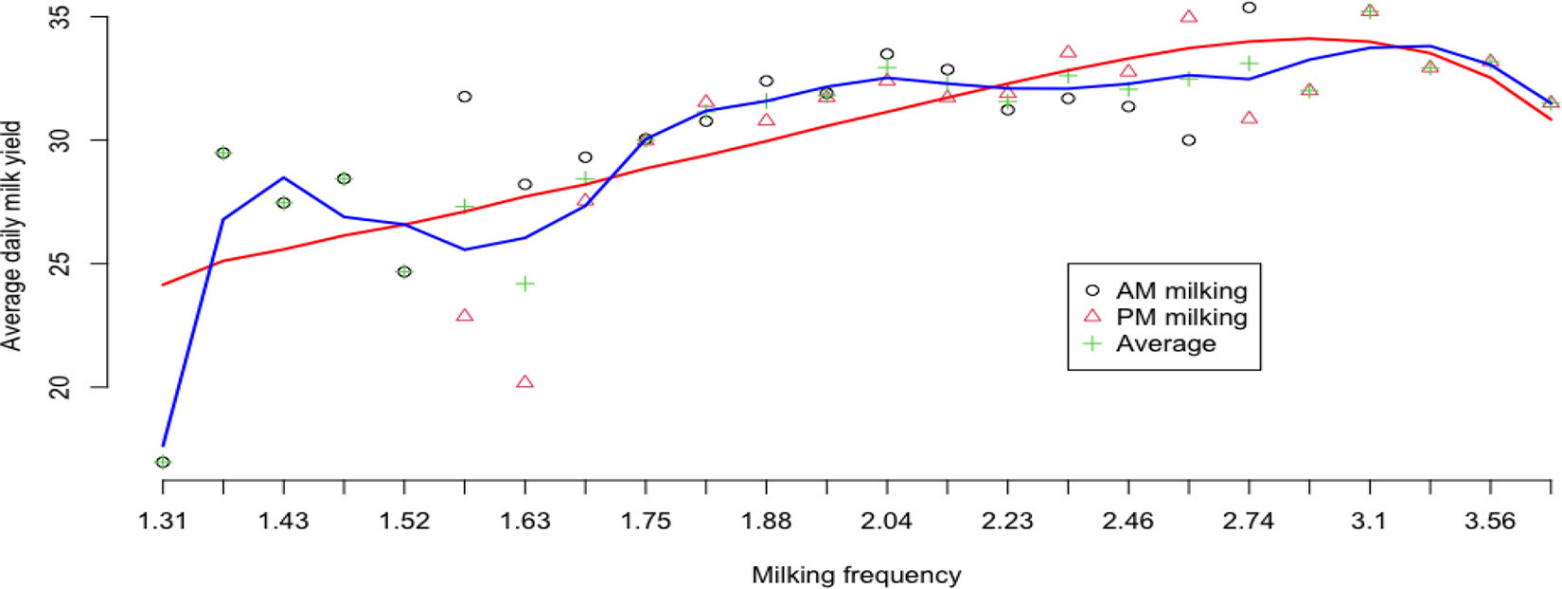
Illustration of average daily milk yields across varying milking frequencies. The red line represents a cubic smoothing, and the blue line shows a locally estimated scatterplot smoothing (LOESS), both fitted to the average daily milk yields calculated from morning (AM) and evening (PM) milkings, respectively, and jointly (average).

We used two population means for 2× daily yields: an average (31.67 kg) of recalculated daily milk yields for cows with milking intervals between 11 and 13 h and an alternatively assumed average of 29.5 kg (Figure 2). The adjustment factors for 1× milking relative to 2× milking were similar between the two models, ranging from 0.716 to 0.743. The adjustment factors for 3× milking were also comparable: between 1.089 and 1.112 with ERF and between 1.054 and 1.091 with PRF. The adjustment factors were roughly similar between the methods, ranging from 1.120 to 1.157 with the ERF model and 1.080 to 1.177 with the PRF model, for milkings between 4× and 10×.

**Figure 2:**
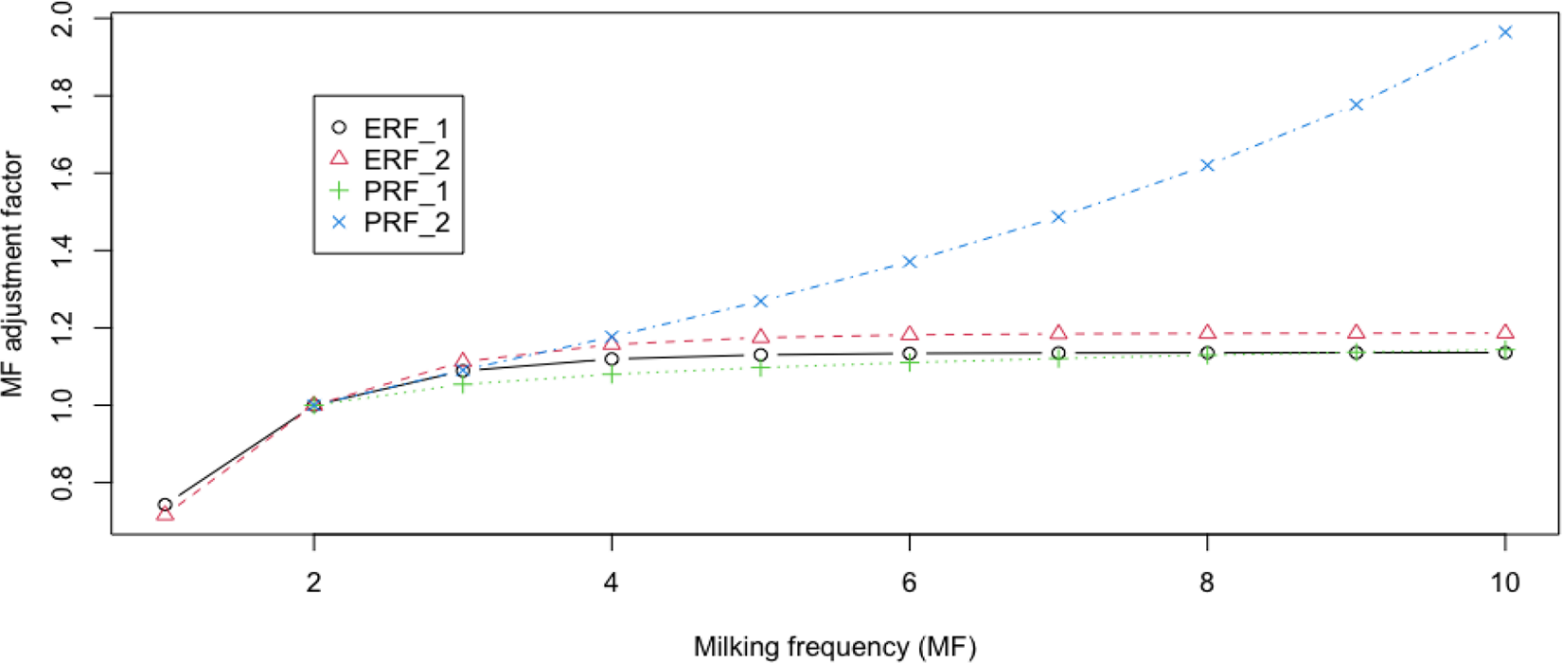
Illustration of predicted milk yield adjustment factors across milking using 2 saturation rational models: ERF = exponential rational function model, and PRF = polynomial rational function model. Both models were fitted on the calculated population means (_1) or an alternatively assumed population mean (_2) for the twice-daily milk yields. MF = milking frequency.

Comparatively, the ERF model yielded more consistent results, indicating that it was more robust to insufficient data than the PRF model. Using a lower-than-actual population mean for 2× daily milk yields resulted in larger adjustment factors with both models. With ERF, milk yield adjustment factors for 5× to 10× milking frequencies ranged from 1.130 to 1.136 using the actual (calculated) population mean, where *c* = 2.89 (0.476), and from 1.175 to 1.186 using the alternative smaller population mean, where *c* = 2.523 (0.327). In contrast, the adjustment factors with PRF increased from 1.097 for 5× milking to 1.143 using the actual larger population mean but showed larger deviations when using the alternative smaller population mean for the 2× daily milk yields. Hence, obtaining an appropriate population mean is crucial for leveraging 2× daily milk yields alone to derive milk yield adjustment factors for milking frequencies. Overall, the adjustment factors estimated from the 2× milking data using the ERF model aligned more closely with results from multiple-milking data than the PRF model. However, caution is advised when extrapolating significantly beyond the available data for higher milking frequencies. In this example, the data extended well only to around 3× or 4× milking, and making projections for higher milking frequencies is less reliable and riskier, particularly with the PRF model.

Technically, applying some restraints may be helpful. For example, based on prior knowledge, one can constrain the average single milk yield to 75% of the average twice-a-day milk yield. For instance, setting *y_t_* = 0.75y_2×_ for *t* = 24, and substituting these values into Equation [6] yields

$$0.75y_{2X}=y_{2X}\frac{{\left(1+a\left(\frac{24}{t}-2\right)\right)}^{b}}{1+c\left(\frac{24}{t}-2\right)}.$$

Simplifying the expression gives

[7] $$a=1-\sqrt[{b}]{0.75\left(1-c\right)}.$$

If we substitute Equation [7] into Equation [6], it then gives a constrained PRF model with 2 unknown parameters:

[8] $$F=\frac{{\left(1+\left(1-\sqrt[{b}]{0.75\left(1-c\right)}\right)\left(\frac{24}{t}-2\right)\right)}^{b}}{1+c\left(\frac{24}{t}-2\right)}.$$

The above illustrates the flexibility of the 3-parameter PRF model. Similar restraints can also be imposed through the relationships between parameters or by adding additional weights to the objective functions. However, such restraints should be taken with extreme caution because they may complicate the model fitting in a not immediately evident way.

Last, the methodologies are described in the context of different milking frequencies based on test-day milk yields. They apply to adjust lactation yields assuming equal adjustment factors across the entire lactation period: $F_{1}=...=F_{m}=F.$ When the milk yield adjustment factors vary across test days, these methods are still valid when calculated from aggregated lactation yields or as a weighted average of adjustment factors across test days or all DIM. In the United States, for example, lactation yields are predicted from test-day milk yields using best prediction (VanRaden, 1997). In the latter case, non-test-day milk yields are imputed from test-day milk yields via the best prediction approach, and a lactation yield is obtained by aggregating daily milk yields for up to 305 d.

Consider the aggregation approach where the lactation milk yield is obtained as a sum of all observed and imputed daily milk yields up to 305 DIM. For the 2× milking plan, the lactation milk yield, denoted by *y*_2×_, is given by

[9] $$y_{2\times }=\sum_{t=1}^{305}x_{t\left(2\times \right)},$$

where *x_t_*_(2×)_ stands for a total daily yield from 2× milking on the *t* DIM. Similarly, the lactation yield calculated based on a 3× milking plan is the following:

[10] $$y_{3\times }=\sum_{t=1}^{305}x_{t\left(3\times \right)},$$

where *x_t_*_(3×)_ stands for a total daily yield from 2× milking on the *t* days in milk. Next, let *F_t_* be an adjustment factor on day *t* in milk when converting the thrice-daily milk yields to twice-daily milk yields, such that

[11] $$x_{t\left(3\times \right)}=F_{t}x_{t\left(2\times \right)}.$$

Then, we can show thrice-milking lactation yields can be converted to twice-milking yields as a weighted average of adjusted daily milk yields through the following relationship:

[12] $$y_{2\times }=\sum_{t=1}^{305}x_{t\left(2\times \right)}=\sum_{t=1}^{305}\frac{x_{t\left(3\times \right)}}{F_{t}},$$

where the weight is *w_t_* = 1/*F_t_*. In the simplest situation where all the adjustment factors are equal across the 305 lactation days (*F_t_* = *F*), then Equation [12] becomes

[13] $$y_{2\times }=\frac{1}{F}\sum_{t=1}^{305}x_{t\left(3\times \right)}=\frac{1}{F}\times y_{3\times }.$$

In conclusion, this study evaluated two rational function models for deriving adjustment factors across varying milking frequencies. Both models performed well in data fitting and predicting unobserved adjustment factors. The PRF model offers enhanced flexibility with its 3 parameters, making it adaptable to a broader range of real-world scenarios. It showed slightly superior fit and accuracy over the EPF model when sufficient data support was available. In contrast, the EPF model proved more robust in the case of limited data coverage across varying milking frequencies. Our findings also indicated that deriving milking frequency adjustment factors is feasible using commonly available 2× daily milking datasets, but caution should be exercised when extrapolating far beyond the data range. Deriving adjustment factors for varied milking frequencies using 3× milking data may also be possible but is not recommended due to its narrow coverage of hypothetical milking frequencies. Finally, the focus of this study was mainly placed on adjusting yields between twice and thrice milkings, which are commonly practiced. Still, further efforts are needed to collect data for optimizing and validating adjustment factors with higher milking frequencies because robotic milking systems are becoming popular.
